# Cardiovascular Findings in Severe Malaria: A Review

**DOI:** 10.5334/gh.789

**Published:** 2020-11-04

**Authors:** Gavin Wooldridge, Deipanjan Nandi, Yamikani Chimalizeni, Nicole O’Brien

**Affiliations:** 1 BC Children’s Hospital, Vancouver, CA; 2 Blantyre Malaria Project, MW; 3 Nationwide Children’s Hospital, Ohio, US; 4 Queen Elizabeth Central Hospital, Blantyre, MW

**Keywords:** severe malaria, echo, cardiac function, hemodynamics

## Abstract

**Background:**

Severe malaria remains a leading cause of death worldwide. A greater understanding of its impact on multiple organ systems is essential in reducing the burden of disease. In this review we will summarize previously reported cardiovascular parameters of both adults and children with severe malaria.

**Method:**

For this systematic review we searched MEDLINE and PUBMED for all papers published on cardiac function in severe malaria from January 1, 1990 until September 1, 2019. Severe malaria was defined as per World Health Organization. Publications were included if there was data from echocardiography, Pulse Contour Cardiac Output (PiCCO), or Pulmonary Arterial catheters (PAC) reported. Studies were excluded if related to medication induced cardiac dysfunction, malaria in pregnancy, or included subjects with known pre-existing heart disease.

**Results:**

Twenty-four studies met inclusion criteria, the majority of which were studies of adult patients or a mixed cohort. Six solely involved pediatric patients. Significant heterogeneity existed in the cardiac parameters measured and results reported. One pediatric and one adult study suggested a reduced preload state during severe malaria. Cardiac systolic function was reported primarily within, or above, normative numeric ranges established in uninfected pediatric patients without anemia. Extensive variability existed in adult studies with reports of an elevated cardiac index in two studies, normal cardiac function in two studies, and descriptions of decreased function in two studies. Two reports suggest afterload in pediatric severe malaria is reduced. Reports of changes in the systemic vascular resistance of adults with severe malaria are inconsistent, with two trials demonstrating an increase and two suggesting a decrease. Studies demonstrated a mild rise in pulmonary pressure in both pediatric and adult patients that normalized by discharge.

**Conclusion:**

Based on limited data, the cardiovascular effects of severe malaria appear to be heterogeneous and vary depending on age. Further detailed studies are required to explore and understand the overall hemodynamic effects of this high burden disease.

## Introduction

Despite the burden of malaria falling worldwide, progress in outcomes has stalled with over 400,000 individuals dying annually. A majority of these deaths occur in young children under the age of five [1]. Severe Malaria (SM) affects multiple organ systems, with frequent respiratory, cardiac, renal and neurological manifestations. Cardiovascular abnormalities in SM have long been appreciated [2] and take a multitude of forms [3,4,5,6,7]. Systemic inflammatory effects due to cytokine release, as well as extensive sequestration of the parasitized red blood cells causing microvascular obstruction in the coronary vessels, are likely contributors to the reported hemodynamic abnormalities [8,9].

In the ‘Mortality after fluid bolus in African children with severe infection’ (FEAST) trial, significantly increased mortality was noted in children who received 20–40 ml/kg of either 5% albumin or normal saline boluses [10]. Those with WHO defined shock at the time of randomization had a substantially increased absolute mortality risk (28%) with fluid bolus therapy. On later analysis, the excess mortality due to fluid bolus was reported to be caused by cardiogenic shock in all subgroups, including those with severe malaria [11]. This conclusion was based on the presence of clinical signs of shock at the point of demise. No objective measurements of cardiovascular function were performed in the FEAST trial. It remains unclear if previous studies that measured cardiovascular parameters directly support the theory that cardiovascular failure is a major contributor to mortality in SM either before or after fluid bolus administration.

We therefore undertook this systematic review of previously published manuscripts describing cardiovascular parameters measured directly during SM infection in pediatric and adult patients. Our aim was to summarize the results in an attempt to improve the overall understanding of the effects of SM on the cardiovascular system.

## Methods

This review was performed according to the Preferred Reporting Items for Systematic Reviews and Meta-Analyses (PRISMA) guidelines [12].

### Data sources and Search Strategy

A search was undertaken of Medline and Embase for studies published up until Sept 2019. Search terms included a combination of the following: ‘malaria,’ ‘cardiac,’ ‘cardiovascular,’ ‘hemodynamic,’ ‘echocardiography,’ ‘ultrasound,’ ‘heart’ and ‘myocardial.’ Relevant references cited in eligible studies were also sought.

### Study Selection

Studies involving either pediatric or adult patients with severe malaria and documented echocardiography or other hemodynamic monitoring parameters published in English were considered. SM was defined as per World Health Organization (WHO) definition [13]; presence of plasmodium falciparum asexual parasitemia and no other confirmed cause for the patient’s symptoms or signs, accompanied by one or more clinical or laboratory features. Papers reviewing the pharmacodynamics of or cardiotoxicity from anti-malarial drugs or vaccines were excluded. Studies related to malaria in pregnancy or those involving patients with known cardiac disease were also not included. Studies whose primary focus was on myocardial infarction/heart failure in adults with SM were excluded given the high probability that the results were secondary to cardiovascular complications in the setting of coronary disease exacerbated by fever and anemia rather than a direct effect of malaria on the cardiovascular system. Those studies describing the effect of malaria on the cardiac rhythm or electrocardiogram were outside the scope of this review.

### Data extraction and synthesis

Data extraction was performed using a standardized form by one review author (GW). The following data was retrieved: author details, year and journal of publication, patient demographics, clinical cardiovascular and echocardiography findings, and results of any invasive cardiac output monitoring including Pulse Contour Cardiac Output (PiCCO) or Pulmonary Arterial catheters (PAC). The findings were then placed into broad categories, including pediatric or adult (some of which included a small number of children) studies and separated by parameters studied.

The methodological quality of the studies included was not assessed.

## Results

**Figure 1:**
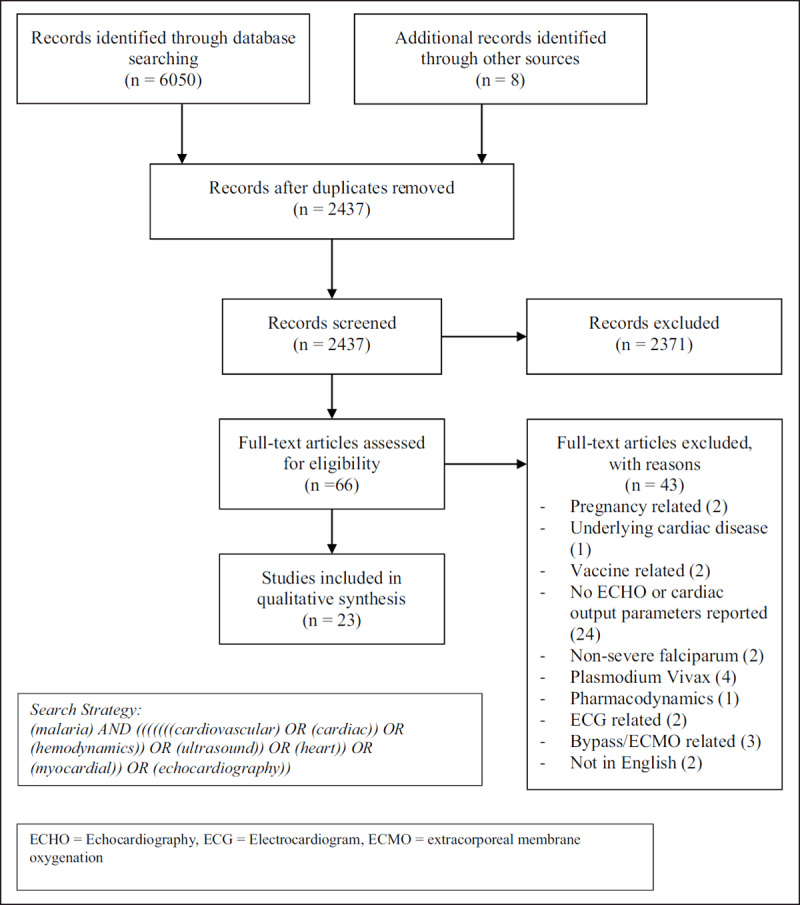
PRISMA Flow Diagram.

6058 citations were identified (Figure 1). After removal of duplicates, 2437 publications remained with 2371 of these excluded after abstract review. Full text review of 66 studies was undertaken, with exclusion of 42 for the following reasons: pregnancy related (2), no echocardiography or cardiac output parameters reported (24), only electrocardiogram (ECG) changes reported (2), underlying cardiac pathology (1), cardiac bypass or extracorporeal membrane oxygenation (ECMO) related (3), non-severe malaria (2), severe plasmodium vivax (4), vaccine related (2), not in English (2) and only pharmacodynamics reported (1). Twenty-three studies met inclusion criteria and are listed in Table 1.

**Table 1:** Descriptive characteristics of all included trials.

Trial	Type of Study	Location	Patient Population	No of participants	Study used to assess cardiac function

Charoenpan (*1990*) [14]	Prospective Cohort	Thailand	Adult with SFM	13	Echo and PAC
Beards (*1994*) [15]	Case Report	South Africa	Adult with SFM undergoing exchange transfusion	1	PAC
Bruneel (*1997*) [16]	Retrospective study	France	Adult with SFM and shock	14	PAC
Lagudis (*2000*) [17]	Case Series	Brazil	Adult	2	PAC and ECHO
Saissy (*2000*) [18]	Prospective observational	Senegal	Adult with SFM	29 (control group=systemic vascular resistance of 800 dyne s^–1^ cm^–5^ or higher. Hyperkinetic group with a level lower than this)	PAC
Mohsen (*2001*) [19]	Case Report	UK	Adult with SFM	1	Echo, cardiac biomarkers
Janka (*2010*) [20]	Prospective observational	Mali	Pediatric with SM and those without. 1–5yrs with SM (excluded if CM though)	53 with SM	Echo, cardiac biomarkers
Yacoub (*2010*) [21]	Prospective observational as part of interventional trial	Kenya	Pediatric with severe malaria (>6m)–SM with metabolic acidosis, children without	30	Echo
Hanson (*2011*) [22]	Prospective observational	Bangladesh and India	Adult with SFM admitted to ICU	28 (same cohort of patients as in Hanson 2013 paper below)	CVP and PiCCO, (Transpulmonary thermodilution)
Herr (*2011*) [23]	Prospective Case control	Germany	Adult, complicated and uncomplicated FM	28	Non-invasive method based on the re-breathing technique
Mocumbi (*2011*) [24]	Prospective observational	Mozambique	Pediatric, 5–15yrs with FM	47, 10 with SM	Echo
Murphy (*2011*) [25]	Pilot observational	Uganda	Pediatric, SFM and non-severe	17 with SM	Echo
Nguyen (*2011*) [26]	Retrospective analysis of prospectively collected hemodynamic data from interventional trials	Vietnam	Adults with SFM	43 (managed with fluid loading or inotropes)	PAC
Sanklecha (*2011*) [27]	Case Series	India	Pediatric	3 (cardiac involvement in one only)	Echo
Nguah (*2012*) [28]	Prospective observational	Ghana	Pediatric	183	Echo
Hanson (*2013*) [29]	Interventional	Bangladesh and India	Adult	28	PiCCO (transpulmonary thermodilution)
Nayak (*2013*) [30]	Prospective observational	India	Adult and Pediatric (13–75yr), severe vivax and SFM	100 with SM, 28/100 with SFM. 9/28 had cardiac involvement	Echo
Sulaiman (*2014*) [31]	Case Report	Malaysia	Adult with SFM	1	Echo
Colomba (*2017*) [32]	Case Reports	Italy	Adults with SFM	2	Echo
Ray (*2017*) [33]	Prospective observational	India	Adult > 15 < 70yr with SFM	23/27 SFM. 7 had circulatory failure	Echo
Kotlyar (*2018*) [34]	Prospective observational–comparison of SM and SMA	Uganda	Pediatric with SFM (3m–12yr)	13 with SM	Echo and cardiac biomarkers (trop I and BNP)
Leopard (*2018*) [35]	Prospective observational–sepsis and SM	Bangladesh	> 12 years, SFM or sepsis	102, 13 with SFM	Lung ultrasound
Kingston (*2019*) [36]	Prospective observational	Bangladesh and India	Adult with SFM or sepsis	46 with SM	ECHO and Expired gas collection

SFM = Severe falciparum malaria, Echo = Echocardiogram, PAC = Pulmonary Arterial Catheter, CVP = Central Venous Pressure, PiCCO = Pulse Contour Cardiac Output, SMA = Severe Malaria Anemia.

### Study Findings: Pediatric

A total of seven studies included in the review evaluated pediatric patients only. All took place in Sub-Saharan Africa. The majority of cardiovascular parameters evaluated are available in Table 2.

**Table 2:** Selected clinical and cardiovascular findings in pediatric studies.

Trial	Mean Age (months)	Mean Hb (dg/L)	Preload D0	Cardiac Function		Structural	Pulmonary Artery Pressures mmHg	Cardiac Biomarkers

				LVEF D0	CI (l/min/m_2_) DO			

Janka (*2010*) [20]	30.1	4.2	–	64%	–	–	31 TRV = 2.5m/s (controls 2m/s)	CK-MB 4.31 ng/mL and Troponin T 10 pg/mL
Yacoub (*2010*) [21]	46(median)	7.5 (median)	IVC collapsibility index 43.8 LVEDD 3.17	63.1%	4.6 Ultrasound Cardiac Output Monitor (USCOM) stroke volume index improved after fluid bolus in 80% of acidotic patients from an average of 36.7 mL/m2 (95% CI, 30.9–42.5) to 41.5 mL/m2 (95% CI, 37.19–45.8; p = .007)	–	–	–
Mocumbi (*2011*) [24]	84	9.3	–	“Preserved systolic and diastolic function”	‘Left ventricular dimensions indexed for body surface were abnormal in two children with severe anemia’(4.4%)	–	–	cTNT undetectable
Murphy (*2011*) [25]	36	7	–	“Good” LV function	–	No pericardial effusions seen	2 patients demonstrated a low-velocity, tricuspid, regurgitant jet (1.5 m/sec and 2.4 m/sec. 3 trace TR. Nil had RV enlargement	–
Sanklecha (*2011*) [27]	Case report of 3 patients with Myocarditis 24,120, 144 of age	7.2	–	–	Patient two only: Myocardial dysfunction with serial ejection fractions of 45%, 35% and 25%	–	–	–
Nguah (*2012*) [28]	36 (median)	7.4	LV-EDDI (mm/m2) 53.15	66%	5.8	–	–	–
Kotlyar (*2018*) [34]	19.2 (median)	5.12		58%	6.4 (T0 all), SM 5.28 T0, SMA 6.89 T0	–	–	Trop I 0.08 (ng/mL) BNP 69.1 (pg/mL)

Data presented as means unless otherwise indicated. Blank = no value given, HR = heart rate, MAP = mean arterial pressure, SMA = severe malaria anemia, LVEF = Left ventricular ejection fraction, CI = Cardiac Index, TRV= Tricuspid regurgitant velocity, LV-EDDI = left ventricular end diastolic diameter index, cTNT = Cardiac Troponin.

#### Significant findings from included pediatric studies related to preload

Multiple studies in pediatric patients demonstrated indices indicative of decreased preload in infected patients:

- Left ventricular end-diastolic diameter index (LV-EDDI), which can be a marker of preload, was slightly lower upon presentation (53.15 mm/m^2^) than at follow up (53.20 mm/m^2^, p = 0.028) [28].
- Left ventricular end diastolic diameter (LVEDD) tended to be lower on admission (3.17 cm ± 0.4) and improved with time (3.27 cm ± 0.33 on discharge, p = 0.47) [21].
  - Normative values for LVEDD for healthy children are 3.5 cm–5.6 cm.
- The inferior vena cava collapsibility index (IVCCI), an indirect marker of preload, was higher for all children on admission (43.8 ± 19.5), than at the time of discharge (21.7 ± 9.8) and was significantly worse in children with acidosis (52.1 ± 21.9) [21].

#### Significant findings from included pediatric studies related to cardiac function

Markers of cardiac function were variable in different studies, using heterogenous timepoints, markers and indices of function:

- Left ventricular ejection fraction (EF) was preserved in all participants [20,25].
  - EF 64% (60–65) in SM and EF 65% (58–65) in controls [20].
  - Normative values for EF for healthy children are >55%.
- Minimal difference in EF was demonstrated in those with varying degrees of severity of illness or anemia. EF 58% (53–62) at admission and EF 54% (51–58) at 24 hours, p = 0.525 [34].
- Nguah found the EF and fractional shortening to be reduced on day 0 compared to follow up but was still within normal range (66% ± 0.06 on admission, 67% ± 0.04, p = 0.008) [28].
  - There was no difference between acidotic and non-acidotic patients on admission or discharge
  - Those with hypotension upon presentation were not studied [21].
- 100/104 had normal EF upon admission [34]
- Left ventricular end systolic volume index (LV-ESVI), reflecting global left ventricular function, was comparable on day 0 (21.7 ml/m^2^ ± 7.1) to the follow up LV-ESVI on day 42 (22.0 ml/m^2^ ± 7.4) [28].
- Cardiac index (CI), stroke index (SI) or velocity time integral (VTI), all surrogates for stroke volume, were all markedly increased during the disease process and decreased after recovery [28,34].
  - CI–5.8 l/min/m^2^ ± 1.8 on day 0 and 4.7 l/min/m^2^ ± 1.4 on follow up [28], median 6.4 l/min/m^2^ (5–7.6, p = 0.001) on day 0 and median 5.46 l/min/m^2^ (4.3–6.6) at 24 hours.
  - Normative values for CI for healthy children >5yrs are 3–4.5 l/min/m^2^.
  - SI–40.8 ml/m^2^ ±10.5 on day 0 and 29.1 ml/m^2^ ± 7.6 on day 42 [28].
  - Normative values for SI for healthy children are 35–65 mls/m^2^.
  - VTI–18.4 (15.8–21.4) on day 0 and 19.2 (17.4–21.3) [34].
  - Normative values for VTI for healthy children are 18–22 cm. The singular risk factor for an increased CI was severe malaria anemia (SMA, defined as a hemoglobin <5g/dL).
    - This was a negative correlation, increase in CI for a decrease in [hemoglobin], with Pearson Correlation Coefficient equal to –0.380 (p < 0.001) [34].
    - CI 6.4 l/min/m^2^ with SMA vs 5.4 l/min/m^2^ without SMA, p ≤ 0.001. LVEDDI 57 mm/m^2^ in SMA vs 51.5 mm/m^2^ without SMA, p ≤ 0.001 [28].
    - Authors concluded their findings suggested an increased cardiac output occurred from a raised stroke volume, with subsequent normalization following transfusion and anti-malarial treatment.
- No evidence of septal flattening in systole or diastole [25], indicating normal pressures of both the right and left ventricle.

#### Significant findings from included pediatric studies related to afterload

While no study directly measured SVR or other parameters of afterload, mean arterial pressure may have been lower in more ill children or those earlier in the time course:

- Mean arterial pressure (MAP) was lower in children with SMA than in those with SM [34].
  - 65.7 mmHg (±18.4) in SMA and 73.5 mmHg (±10.9) in SM.
- Systolic blood pressure and MAP increased from admission (64.3 mmHg ±12) to day 42 follow up (73.3 mmHg±8) [28].

#### Significant findings from included pediatric studies related to pulmonary artery pressures

Pediatric patient pulmonary arterial pressures were higher than control patients:

- Janka reported an increase in pulmonary arterial pressures (mean PAP = 31 mmHg) in comparison to controls (mean PAP = 21 mmHg) [20].
  - No potentially causative left ventricular dysfunction was seen.
- Murphy stated that no right ventricular enlargement was seen in her patients, and only three of 26 had trace tricuspid regurgitation [25].

#### Significant findings from included pediatric studies related to structural alterations

No studies mentioned the presence of pericardial effusions or other structural abnormalities.

#### Significant findings from included pediatric studies related to cardiac biomarkers

Troponin subtypes varied from normal to elevated in some studies, while BNP and NT-proBNP levels were higher in the two pediatric studies that followed it:

- Troponin T and CK-MB levels were the same as in control patients [20].
- Forty-eight percent of all children (n = 50) had elevated levels of Troponin I (cTnI).
  - Raised in 42% of SM (n = 18) and 53% of SMA patients (n = 32) [34].
  - Normative values of Troponin I in healthy children are < 0.1 ng/ml.
- Cardiac Troponin T was undetectable (levels below 0.03 ng/mL) in any child with severe or complicated malaria [24].
- SM cases had higher plasma *NT*-proBNP levels than controls [20].
  - NT-proBNP improved prior to discharge.
  - Normative values of BNP in healthy children are < 100 pg/mL.
- Nineteen percent of all children (n = 20) had mildly elevated levels of BNP at Time 0 [34].
  - Seven percent of SM patients (n = 3) and 28% of SMA patients (n = 17).

### Study findings: Adults

Sixteen studies included in the review evaluated a majority of adult patients. Studies took place worldwide. Table 3 reports studies describing echocardiography findings in patients with myocarditis, pericarditis, myocardial ischemia and cardiac dysfunction thought to be primarily related to SM. Table 4 represents selected clinical and measured cardiovascular findings in adult studies reporting invasive measurements of cardiac output.

**Table 3:** Adult echocardiogram findings of myocarditis, pericarditis or myocardial ischemia.

Trial	Proposed SM induced cardiac diagnosis	Age (years)	Hemoglobin g/dL	Dimensions	EF %	Structural	Pulmonary artery pressures mmHg	Cardiac Biomarkers

Mohsen (*2001*) [19]	Myocarditis	30	11.2	–	D0 admission echo normal. Cardiac Output was supra-normal at 11l/min. Echo on d10 demonstrated severe global left ventricular dysfunction with no regional wall abnormalities (EF 38%)		Normal RAP and PWP (12–18mmHg)	Normal creatinine phosphokinase
Nayak (*2013*) [30]	–	13–75years	–	(LVEDD) of 4.04 (LVESD) of 2.55	56% with cardiac involvement (59% without cardiac involvement)	9 patients had mitral regurgitation, mild tricuspid regurgitation, mild aortic regurgitation and mild pulmonary regurgitation; these findings were present at the time of admission, on the day of discharge as well as on Day 21 of follow up. None of these patients had any valvular thickening. No patient had any evidence of pericardial effusion and regional or global hypokinesia		Both Troponin-I and CPK-MB were increased in 14% cases and were found normal in 3 out of 17 patients who presented with cardiovascular involvement
Sulaiman (*2014*) [31]	Myocardial Ischemia	51	10.7	–	Hyperdynamic contractility with preserved LV systolic function			Normal (and normal coronary angiography)
Colomba (*2017*) [32]	Pericarditis	19 and 52	8.2/8.6	–		Pt 1. Revealed an anterior non-compressive pericardial effusion (6 mm behind the right atrium, 9 mm in lateral) and a congenital intra-atrial and intra-ventricular communication with left-to-right shunt Pt 2. Left ventricular concentric hypertrophy with preserved global systolic function, absence of any segmental wall-motion abnormalities of the left ventricle; right sections were of normal size with preserved right ventricular function. It also showed pericardial effusion		-
Ray (*2017*) [33]	–		–	–	<55% in 3, left ventricular diastolic dysfunction in 1	mild pericardial effusion (1)	mild TR with mild PAH (1)	–
Leopard (*2018*) [35]	–	33		LVFS % 41 IVC collapsibility % 18 Uncomplicated 31% LVFS 26% IVCC Sepsis 31% LVFS 26% IVCC (all medians)	–			–

Data presented as means unless otherwise indicated. Blank = no value given, LVEF = Left Ventricular Ejection Fraction, LVEDD = Left Ventricular End Diastolic Diameter, LVESD = Left Ventricular End Systolic Dysfunction, TR = Tricuspid Regurgitation, PAH = Pulmonary Arterial Hypertension.

**Table 4:** Adult invasive cardiac output monitoring findings.

Trial	Invasive Monitoring	Preload: CVP	Cardiac Index (L/min/m^2^)	SVR (dyne/s/cm^–5^m^2^)	PAOP mmHg	Cardiac Biomarkers

Charoenpan (*1990*) [14]	PAC	–	4.66	832 reported as low as (normal values 900–1100 in paper) low PVR	–	–
Beards (*1994*) [15]	PAC	12 (prior to exchange)	4.42	586	–	–
Bruneel (*1997*) [16]	PAC–7 patients only	–	–	Peripheral vasodilatation with elevated cardiac output	–	–
Lagudis (*2000*) [17]	PAC – hyperdynamic pattern and normal LV stroke work index	Normal echo	1^st^ patient 6 2^nd^ patient 4.3	SVRI 1^st^ patient 1049 2^nd^ patient 1078	1^st^ patient 17 2^nd^ patient 15	–
Saissy (*2000*) [18]	PAC		3.9 control group 6.1 hyperkinetic group	1098 control group 536 hyperkinetic group	6 control group 9 hyperkinetic group	–
Hanson (*2011*) [22]	CVP and PiCCO	5.2 (median)	3.08 (median)	SVRI 2155 (median)	–	–
Herr (*2011*) [23]	Non-invasive method based on the re-breathing technique	-	2.9 9SM cases (healthy controls 3.4) (median)	SVRI 29.2 l/min (median)	–	Pro-BNP 139.3 pg/ml Myoglobin 43.6 μg/l Trop T and CK-MB similar to controls H-FABP 1.9ng/ml (1.7 in uncomplicated)
Nguyen (*2011*) [26]	PAC	2 fluid load, 4.5 no fluid load	4 (with and without fluid load)	1633/without fluid 1589	6 fluid load/10 no fluid loading	–
Hanson (*2013*) [29]	PiCCO	4.5 (median)	3.08 (median)	2155 (median)	–	–
Kingston (*2019*) [36]	ECHO and Expired gas collection	–	4.1 (median)	–	–	–

Data presented as means unless otherwise indicated. SFM = Severe falciparum malaria, Echo = Echocardiogram, PAC = Pulmonary Arterial Catheter, CVP = Central Venous Pressure, PAOP = Pulmonary Artery Occlusion Pressure, PiCCO = Pulse Contour Cardiac Output, SMA = Severe Malaria Anemia, SVR = Systemic Vascular Resistance, SVRI = Systemic Vascular Resistance Index, DO2 = Oxygen delivery, VO2= Oxygen consumption. NT-proBNP = N-terminal pro-brain natriuretic peptide; CK-MB = creatine kinase-muscle brain; TnT = troponin T; H-FABP = heart-type fatty acid-binding.

#### Significant findings from included primarily adult studies related to preload

Adult studies of preload found varying markers:

- Fluid loading resulted in a rise in cardiac index and oxygen delivery [26] with a mean change of 0.75 l/min/m^2^ in CI (–.41 to 1.1) and 26 ml/min/m^2^ (–2 to 54) in DO_2_.
  - MAP and CVP changed by 2 (–1 to 6) and 3 mmHg (1–4) respectively with fluid loading.
- LVEDD was 4.04cm ±0.4 in those with cardiac involvement (defined as the presence of circulatory failure, congestive heart failure or pulmonary edema) compared to those without having LVEDD of 3.76 cm ± 0.43, p ≤ 0.0001 [30].
  - Normative values of LVEDD in healthy adults are 3.5–5.7 cm.
- LVESD was 2.55 cm ±0.44 in those with cardiac involvement compared to those without, LVESD of 2.45 cm ± 0.34, p ≤ 0.0001 [30].
- Hanson found a median (range) Global End Diastolic Volume Index (GEDVI, a marker of volume status) on admission of 481 (346–675) [22].
  - Normative values are >680 ml/m^2^.
  - There was no correlation betwe^e^n the baseline CVP and the likelihood of the CI being volume responsive, nor between the change in CVP and change in CI with fluid loading [22].

#### Significant findings from included primarily adult studies related to cardiac function

A majority of adult studies found some degree of cardiac dysfunction or low end of normal, although some studies found higher cardiac function:

- EF was marginally reduced in those with circulatory failure and pulmonary edema (56.34 ± 1.04 all patients) compared to those without (59.11 ± 1.12 p = 0.01) [30].
  - Nine of 28 SFM cases had cardiac involvement and was found to be more common than in P. Vivax (p < 0.001).
- Hanson reported the median CI for the entire cohort was 3.1 (2.27–5.24 l/min/m^2^) and was thus considered normal although 10 patients had a CI less than 3 [22].
- When using an expired gas collection model, CI was increased in SM, 4167 ml/min/m^2^ median (3564 to 4876 ml/min/m^2^) [36].
  - Healthy volunteers in this study had a CI of 2575 ml/min/m^2^ (2340 to 3111 ml/min/m^2^).
- Herr discovered that CI and SI were reduced in malarial patients compared to controls (CI: 2.7 l/min/m^2^ versus 3.4 l/min/m^2^, P < 0.001; SI 31.3 ml/m^2^ versus 43.6 ml/m^2^, P < 0.001, respectively) [23].
- Mean cardiac index was increased > 4l/min/m^2^ in both pulmonary edema and non-pulmonary edema groups [14].
- Significant increases in extravascular lung water occurred in 17 of 22 (77%) patients who were liberally resuscitated, with eight developing frank pulmonary edema despite being hypo- or euvolemic [29].

#### Significant findings from included primarily adult studies related to afterload

Studies had conflicting results with regards to measures of afterload in adult patients:

- Mortality was higher in the 50% of patients in the Hanson’s series who developed severe generalized edema. The GEDVI fell in the first 24 hours in all patients who died despite continuing fluid administration [29].
  - Authors concluded this represented indirect evidence of reduced SVR and increased systemic vascular permeability.
- The change in SVR reported with fluid loading was –242 dyne/s/cm^–5^m^2^ (–380 to –104 dyne/s/cm^–5^m^2^) [26].
  - There was no significant difference between the CVP of the patient who did and did not have pulmonary edema on admission. There was no relationship between the baseline CVP and the volume of fluid that was required to resuscitate the patient.
- Significantly elevated systemic vascular resistance index (SVRI) was found in SM patients [23]. (Cases: 32.6, controls 23.2 dyne/s/cm^–5^m^2^, P < 0.001).
- SVR was low prior to exchange transfusion in one case report (586 dyne/s/cm^–5^m^2^) [15].
- SVR was reported to be low early in the disease course, (832 dyne/s/cm^–5^m^2^) [14].
  - Normative value of SVR in healthy adults is approximately 800–1200 dyne/s/cm^–5^m^2^.

#### Significant findings from included primarily adult studies related to afterload pulmonary artery pressure (PAP)

- Mild TR and mild pulmonary arterial hypertension (PAH) identified [33].
- PVR was found to be low and persistent throughout the study [14].

#### Significant findings from included primarily adult studies related to structural alterations

Pericardial effusion was a rare finding in adult studies: No patient had any evidence of pericardial effusion or regional hypokinesia [30].

- One patient of 27 in one study [33] and two case reports [32] of SM induced pericarditis had evidence of a pericardial effusion.

#### Significant findings from included primarily adult studies related to cardiac biomarkers

Cardiac biomarkers also varied between studies:

- Herr demonstrated that Troponin T was not raised in malarial cases compared to healthy controls [23].
- H-FABP and myoglobin were almost twice those of controls in patients with SM (H-FABP: 1.9 ng/ml ± 1.1, and in controls 1.1 ng/ml ± 1.6, p < 0.001. Myoglobin 43.6 mg/l ± 12.5 and in controls 27.8 ±15.0, p =< 0.001) [24].
  - NT-proBNP and CK-MB were not significantly elevated [23].
- Cardiac markers, both Troponin-I and CK-MB were increased in 14% cases and were found normal in 3 out of 17 patients who presented with cardiovascular involvement [30].

## Discussion

Improved understanding of the cardiovascular effects of SM is an imperative step in the identification of appropriate adjunctive therapies that may reduce the morbidity and mortality of this global health menace. The results of this systematic review highlight the heterogeneity of reported effects of severe malaria on the cardiovascular system. Numerous reasons may account for the variability in the reported results including the relatively small number of patients evaluated as well as differences in approaches and parameters measured.

Suggestion of a reduced preload state was present in two pediatric studies and one adult study. This would fit the common clinical presentation and suspicion of hypovolemia, especially in children who are commonly unwell for a few days prior to admission with vomiting, fever and a reduced intake. Through the use of tracer dilution methods and bioelectrical impedance, Planche et al. demonstrated that mild dehydration was often present in children with SM (mean (SD) depletion of TBW of 37(±33) ml/kg) [37]. Maitland et al. recruited patients with evidence of compensated shock (tachycardia, prolonged capillary refill and low central venous pressure (CVP) < 5cmH_2_O) and reported an improvement in haemodynamics and acidosis with administration of fluid bolus, concurrently with an increase in CVP [38]. However, other studies do not demonstrate significant preload loss and with the minimal literature available, it is difficult to make definitive conclusions, except that it may be present in some SM cases.

Cardiac systolic function in both pediatric and adult studies was demonstrated to be ‘normal’ or ‘supra-normal’ in a number of studies. However, the finding that there are ‘normal’ cardiac functional parameters does not mean that there is an adequate cardiac output in the face of severe anemia and cytokine induced capillary leak. In two pediatric studies there appeared to be an increase in cardiac index, as expected in those with severe anemia, predominately produced by a rise in stroke volume. A significant proportion of the remaining studies performed echocardiography after the initial resuscitation period. It is therefore difficult to determine what overall effect fluid loading had on cardiac function and if it was normal or reduced prior to the intervention in these studies.

Presumably due to sequestration in the myocardial vessels with secondary ischemic changes, myocarditis with diminished cardiac function is not uncommonly reported in adults with SM [19,39,40]. Similar findings have not clearly been reported in pediatric patients except one included case report [27]. Cardiac biomarkers are largely normal or at the most, modestly elevated, with return to normal following resolution of the acute infection in the presented studies. Myocardial oxygen demand is high during acute SM due to tachycardia, and thus elevations in these values may occur secondary to myocardial stress rather than any significant myocardial injury.

The effect of SM on afterload is unclear as the results evaluating this in the available literature are mixed. In a limited number of pediatric patients, blood pressure tended to increase from admission to follow up in SM patients, perhaps indicating initial reduced afterload. Other adult trials also suggest low to normal SVR. This is in comparison to evidence for increased systemic vascular resistance in two different adult studies. The interaction of the plasmodium infected erythrocytes with the endothelium has been extensively studied. Intuitively, pro-inflammatory cytokine release known to occur in SM should result in reduced systemic vascular resistance [13,14,28,41]. Alternatively, extensive microvascular and widespread obstruction from parasitized and sequestered red blood cells may result in increased SVR [22]. At an individual level, variations in the overall severity and impact of each of these pathophysiological processes may occur in patients with SM. If this is the case, this may explain the differing results of available studies. It is important to note that anti-malarials such as intravenous quinine, are commonly considered cardiodepressants, and their use may be at least partially responsible for some of the cardiovascular effects reported in the literature before artesunate became standard of care.

Approximately 10–15% of children and 10% of adults with severe malaria present with shock [41,42,43,44,45], and those that do have a very high mortality [46]. These patients have traditionally been managed with fluid resuscitation and vasoactive medications to support failing haemodynamics. Additionally, adults have a propensity towards ill-defined and frequently fatal pulmonary edema during the management of SM [29,30,47,48]. Such pulmonary edema associated with a mortality rate of 80% in resource limited settings [48]. One would expect these apparently preload deficient and hypoperfused patients, with mostly normal cardiac function and variable afterload, to respond well to fluid therapy. Indeed, the cardiac function of these shocked patients may not be considered normal in the face of SM and they could actually have myocarditis. It is clear, however, that fluid loading increases mortality in children despite improving perfusion at one hour [10,11] and recent re-analysis of the data discovered that the detrimental effect on mortality risk persisted for up to 4 days post randomization [49]. Fluid loading also increases the risk of pulmonary edema in adults and is associated with worse outcomes [50]. The overall results of the available studies presented here, that directly measure myocardial function, do not support clear mechanisms by which a poor response to fluid resuscitation would occur.

Future research needs to be done in larger numbers of patients meeting strict and uniform inclusion criteria with the same instruments of measurements and parameters recorded. In order to conclusively understand the effect of SM on the cardiovascular system, cardiac function needs to be closely and quantitatively tracked over the disease duration.

## Conclusion

Available studies of children and adults with SM report variable changes in preload, myocardial contractility, and systemic vascular resistance. It remains unclear if there is legitimate heterogeneity in these measurements across a cohort of patients with SM or if findings are limited by patient numbers and disparate approaches to and timing of measurements. Larger, more detailed studies are required to explore and understand the cardiovascular abnormalities in this multi-systemic high burden disease, including if there is a subset of SMA patients with under-recognized cardiac dysfunction despite normal indices.

## Additional File

The additional file for this article can be found as follows:

10.5334/gh.789.s1 Supplementary Material. PRISMA-P checklist.
